# Migraine genetics: from genome-wide association studies to translational insights

**DOI:** 10.1186/s13073-016-0346-4

**Published:** 2016-08-19

**Authors:** Padhraig Gormley, Bendik S. Winsvold, Dale R. Nyholt, Mikko Kallela, Daniel I. Chasman, Aarno Palotie

**Affiliations:** 1 Psychiatric and Neurodevelopmental Genetics Unit, Massachusetts General Hospital and Harvard Medical School, Boston, MA 02114 USA; 2 Medical and Population Genetics Program, Broad Institute of MIT and Harvard, Cambridge, MA 02142 USA; 3 Stanley Center for Psychiatric Research, Broad Institute of MIT and Harvard, Cambridge, MA 02142 USA; 4 FORMI, Oslo University Hospital, PO 4956, Nydalen, 0424 Oslo Norway; 5 Department of Neurology, Oslo University Hospital, PO 4956, Nydalen, 0424 Oslo Norway; 6 Institute of Clinical Medicine, University of Oslo, PO 1171, Blindern, 0318 Oslo Norway; 7 Statistical and Genomic Epidemiology Laboratory, Institute of Health and Biomedical Innovation, Queensland University of Technology, 60 Musk Ave, Kelvin Grove, QLD 4059 Australia; 8 Department of Neurology, Helsinki University Central Hospital, Haartmaninkatu 4, 00290 Helsinki, Finland; 9 Division of Preventive Medicine, Brigham and Women’s Hospital, Boston, MA 02215 USA; 10 Harvard Medical School, Boston, MA 02115 USA; 11 Analytic and Translational Genetics Unit, Massachusetts General Hospital and Harvard Medical School, Boston, MA 02114 USA; 12 Institute for Molecular Medicine Finland (FIMM), University of Helsinki, 00014 Helsinki, Finland; 13 Department of Neurology, Massachusetts General Hospital, Boston, MA 02114 USA

## Editorial summary

Understanding the molecular mechanisms that precede and give rise to a migraine attack is key to developing new therapeutic strategies. Advances towards this goal have recently been made through genome-wide association studies, which have identified new genetic components of migraine that highlight vascular etiologies and underline the polygenic nature of this disorder.

## Understanding the polygenic basis of migraine

As the most prevalent and disabling neurological disorder, migraine affects the lives of millions of people worldwide, and for many there are still no effective treatments. Genome-wide association (GWA) studies are an important approach used to uncover the genetic susceptibility components of complex diseases such as migraine. The most recent GWA study [1], which was conducted by groups including our own, has identified 38 genomic loci commonly found in humans (>5 % allele frequency) that influence migraine risk. Earlier studies [2–4] have implicated additional loci, putting the total number of genomic regions associated with migraine as high as 47. These loci represent great progress in the field and provide cause for optimism that key mechanisms can eventually be understood. However, as is typical in GWA studies, the impact of each individual locus on disease risk is relatively modest (odds ratios <1.2), thus making it difficult (and perhaps not useful) to interpret the contribution of any one implicated gene to migraine pathophysiology. Instead, the power of these genomic loci comes from assessing their contribution as a group, whereby through aggregation in certain pathways or other functional classifications they can provide a map towards biological mechanisms of the disease. In this way, new hypotheses can be generated that can then inform functional studies to reveal the underlying biology.

Part of the problem in understanding the biological underpinnings of migraine has been that there are no known or measurable biomarkers for the disease. Furthermore, the common forms of migraine have been firmly established as polygenic disorders [1–3] in which likely hundreds of genetic variants across the genome accumulate in varying combinations to give rise to the spectrum of disease outcomes. Understanding how these different combinations of variants (along with the multitude of environmental triggers) can influence the level of risk across individuals and produce specific disease outcomes is a complex problem to solve. For instance, it is not clear how genetic variation and triggers may interact, nor is it clear what might be the contribution of rare variation. Answers to these questions will be revealed only with appropriately designed study populations.

An important aspect for a successful study is the sample size, where genotyping larger numbers of cases and controls facilitates, by boosting statistical power, the identification of true risk loci. The most recently published migraine study, comprising data from 375,000 individuals, represents the largest published GWA study of any specific disease [1]. Collecting such numbers is usually only possible through international collaborations that span academic, clinical, and commercial institutions. In this study [1], partnerships with commercial entities were hugely important to increase the sample numbers. For example, out of 375,000 individuals, the companies 23andMe and deCODE together provided data from 56 % (or 33,600) of the total cases and 76 % (or 238,732) of the total controls. These public–private partnerships should be encouraged in future studies as they benefit all interested parties, and the pooling of all available resources can only help speed up new discoveries. Of course, it should be remembered that the contributions from academic or clinical institutions are equally important because these tend to consist of more severe cases and better phenotyped collections that can also improve power and enable more detailed follow-up questions to be investigated [5].

Here we outline the major findings from the latest GWA study of migraine [1] and discuss what they reveal about both the pathophysiology and genetic architecture of the disease. We further discuss the implications of these findings for translational research and clinical treatment.

## Key genomic loci implicated in migraine: a more central role for vascular etiologies

A key finding from the most recent migraine GWA study [1] was that the 38 identified loci were enriched for genes that are expressed most actively in arterial tissue. This is an interesting discovery because although migraine is known to have an impact on vascular function, it was thought that this most likely represented downstream effects from neuronal activity rather than pathology of the vasculature itself. These findings suggest that vascular dysfunction plays a much more central role in migraine pathophysiology and could even be more important than the neuronal component in some individuals. This vascular discovery aligns well with known co-morbidities and shared polygenic risk between migraine, stroke, and cardiovascular diseases [6, 7]. Furthermore, the lead single-nucleotide polymorphism identified in the *PHACTR1* locus for migraine has also been identified as the lead polymorphism associated with several vascular diseases (coronary heart disease, coronary artery calcification, and cervical artery dissection) [1]. For cervical artery dissection, there also appears to be overlap with migraine at two more loci (*LRP1* and *FHL5*) [8], suggesting the possibility of partially shared genetic components between migraine and these diseases.

However, the likelihood that neuronal dysfunction still has an important role in migraine pathophysiology should not be discounted because, although the 38 loci as a group were not found to be enriched in the brain, several individual loci showed strong expression in specific brain tissues. Furthermore, compared with vascular and other tissues, well-characterized brain samples are more difficult to obtain for research purposes and, therefore, perhaps the specific brain tissue most relevant for migraine has not yet been assayed. It is also possible that when more loci are identified through future studies, an enrichment in brain tissues will become clear.

Previous hypotheses of molecular mechanisms in migraine have come from familial hemiplegic migraine (FHM), a rare Mendelian form of the disease, where three ion channel genes are known to be involved (*CACNA1A*, *ATP1A2*, and *SCN1A*) [9]. These findings propagated the theory that more common forms of migraine might also be characterized as channelopathies. However, in the recent study only two ion channel genes were identified out of the entire set of 38 loci (*KCNK5* and *TRPM8*) [1]. This also agrees with earlier studies suggesting that ion channel dysfunction is not the most important pathophysiological mechanism in common forms of migraine [10]. However, genes at three other migraine-associated loci (*SLC24A3*, *ITPK1*, and *GJA1*) have been linked to ion homeostasis, so it is possible that genes more generally involved in this biological process could have a role.

The recent findings [1] are also beginning to reveal the genetic architecture of migraine and its common subtypes: migraine with aura (MA) and migraine without aura (MO). For example, in a subset analysis consisting of individuals with only MO, seven loci were identified, whereas for individuals specifically suffering from MA, no associated loci were found [1]. This seems to suggest that the genetic architecture of these two forms is quite different. However, in a follow-up heterogeneity analysis [1], most of the 38 loci were actually implicated in both migraine subtypes, suggesting that the absence of significant loci for MA is mainly due to lack of statistical power from the lower number of samples available. To add weight to this argument, estimates of heritability (based on linkage disequilibrium score regression) found that the MA dataset captured less of the heritability than the MO dataset, such that approximately twice the sample size would be required to reach equivalent power for MA as was obtained for MO [1]. Possible explanations for these differences are that greater heterogeneity might have been introduced in the clinical phenotyping, that low frequency or rare variation may contribute more to the risk, or perhaps even that the underlying biology of MA is simply more complex.

## Conclusions and clinical implications

Although the advances in GWA studies represent major progress, it is perhaps too early to say whether, in the short term, the common variant loci found could directly help to influence patient care. Certainly, the magnitude of the genetic effects are not sufficient to make predictions of migraine outcomes in individuals, as the proportion of the heritability explained by these loci is, on aggregate, very low. In the longer term, however, there are great opportunities to make improvements by sub-classification of individuals based on their genetic profile and the potential for tailoring treatments specific to individual patients.

Before this, however, appropriate investigation would be required of the genetic factors influencing the variability in response to certain treatments. For example, we still do not understand how the genetic findings are linked to treatment response to triptans (which constrict the blood vessels of the brain), nor to responses to calcitonin gene-related peptide (CGRP) antagonists and CGRP-blocking antibodies (CGRP levels have been found to be raised during migraine attacks in some individuals) [9]. The recent GWA study results [1] have added to the ongoing discussion concerning site of action of these drugs because, although the triptans have both vascular and neuronal effects, the new CGRP antibodies have a molecular size too large to get across the blood brain barrier. The genetic findings therefore provide insight for new study designs, suggesting a revised approach and a focus on vascular influences, that may address questions of individualized treatment responses and potential new treatment options. Moving towards more personalized care is certainly the goal, but getting there will need well-designed studies with larger sample sizes and a deeper understanding of how low frequency and rare variants influence the disease traits.
